# Risk of Death and Recurrent Ventricular Arrhythmias in Survivors of Cardiac Arrest Concurrent With Acute Myocardial Infarction

**Published:** 2008-02-01

**Authors:** Ish Singla, Haitham Hreybe, Samir Saba

**Affiliations:** Cardiovascular Institute, University of Pittsburgh Medical Center, Pittsburgh, Pennsylvania

**Keywords:** Cardiac Arrest, Ventricular Arrhythmias, Myocardial Infarction, Defibrillators, Mortality

## Abstract

**Aims:**

Cardiac arrest (CA) is an indication for defibrillator (ICD) implantation unless it occurs in the context of an acute myocardial infarction (AMI). We investigated the ventricular arrhythmia (VA)-free survival of patients resuscitated from CA in the setting of AMI.

**Methods:**

We reviewed a database of 1600 AMI and CA survivors from which 48 patients were identified as having concurrent CA and AMI (CA+AMI group). Those patients were matched by age, gender, race, and left ventricular ejection fraction (LVEF) to 96 patients with AMI but no CA (AMI group) and 48 patients with CA but no AMI (CA group).

**Results:**

Patients and controls were followed for 3.9±3.2 years. Patients in the 3 groups had similar baseline characteristics (age 63±14 yrs, 78% men, 98% white, 53% with CAD, LVEF 33±14%). The 5-year VA-free survival was 67%, 92%, and 80% for the CA+AMI, AMI, and CA groups, respectively, p<0.001.

**Conclusion:**

Patients with concurrent CA and AMI are at high risk of recurrent VA, with VA-free survival rates significantly worse than those of patients with AMI but no CA, and comparable to those of patients with CA outside the context of an AMI. Accordingly, these patients should be considered for ICD implantation.

## Condensed Abstract

Patients with concurrent CA and AMI were found to be at high risk of recurrent VA, with VA-free survival rates significantly worse than those of patients with AMI but no CA, and comparable to those with CA only. Accordingly, these patients should be considered for ICD implantation.

Cardiac arrest (CA) or hemodynamically compromising ventricular arrhythmias (VA) constitute an indication for defibrillator (ICD) implantation [1-3] except when these life-threatening events occur in the context of acute myocardial infarction (AMI), which is considered a transient or reversible cause [4]. In fact, in this context, the implantation of an ICD is given a class III indication, i.e. it is considered not useful and potentially harmful [4]. There are however very few data to support these guidelines and recommendations.

Data from the AVID registry [5] suggest a high CA recurrence rates amongst survivors of CA whose events were felt to be associated with reversible causes such as myocardial ischemia or infarction. In the current era where ICDs are implanted for primary prevention purposes [6-9], we designed this study to investigate the risk of recurrence of VA or death in survivors of CA in the context of AMI as compared to 2 control groups: survivors of AMI with no CA or VA (AMI group) and survivors of CA outside the context of an AMI (CA group).

## Methods

### Population

All patients who were admitted to the University of Pittsburgh Medical Center with CA concurrent with AMI between 1992 and 2000 were included in this study. Patients electronic medical records were analyzed for baseline demographics, electrocardiographic data, clinical characteristics, left ventricular ejection fraction (LVEF), laboratory data both upon admission and discharge, admission and peak cardiac enzymes, inpatient medications and therapies, discharge medication, echocardiographic data, left and right heart catheterization data, ICD implantation and therapies, and associated medical conditions.

A total of 1600 records were reviewed, of which 48 consecutive patients with concurrent CA and AMI were identified. For each such patient, two controls with AMI but no CA and one with CA outside of the context of an AMI were identified and matched by age, gender, race and LVEF. A total of 192 patients were included in the analysis. All-cause mortality was obtained by reviewing medical records and by searching the National Social Security Death Index [10]. The primary end points of the study was the time to death or first recurrence of sustained VA, defined as a VA requiring hospital evaluation, intervention, or appropriate ICD therapy. The study was reviewed and approved by the Internal Review Board of the University of Pittsburgh.

### Statistical analysis

Continuous variables were expressed as mean ± standard deviation and were compared using the student t-test. Categorical variables were compared using the Chi-square test. Times to event curves during the follow-up period were calculated by the Kaplan-Meier method and compared between groups using the log-rank tests. Multivariate analysis of the independent predictors of VA-free survival was performed using the Cox Proportional Hazards model. A two-tailed p-value of <0.05 was considered significant. All analyses were performed using SPSS 14.0 version (Chicago, IL).

## Results

### Demographic data

A total of 48 consecutive patients were admitted to the University of Pittsburgh Medical Center with concurrent CA and AMI. They were matched to 96 control patients with AMI but no CA and to 48 control patients with CA outside the context of an AMI. Cases and controls were followed at our institution for a mean duration of 3.9±3.2 years. The demographic and clinical characteristics of patients and controls are shown in Table 1.

Compared to the patients in the AMI group, patients in the CA+AMI group were more likely to have a history of coronary artery disease (56.3% vs.36.5%, p=0.018), of CA (8.3% vs. 0%, p=0.004), and atrial fibrillation (25% vs. 3.1%, p<0.001). Compared to patients in the CA group, cases (CA+AMI group) were less likely to have a history of cardiomyopathy (20.8% vs. 58.3%, p=0.001). Also, the use of β-blockers, Angiotensin converting enzyme inhibitors, and anti-arrhythmic medications were higher in the survivors of concurrent CA and AMI compared to the patients in the control groups.

As expected based on published guidelines [4], there was a large discrepancy (p<0.001) in the use of ICD therapy between the 3 study groups, with the survivors of CA outside the context of AMI having the highest rates (98%), followed by survivors of concurrent CA and AMI (73%), followed last by survivors of AMI without CA (7%). It is worth noting that based on the most recent guidelines, which state that patients with LVEF?35% are indicated for ICD implantation, 56 of 96 (58%) patients in the AMI group and 29 of 48 (60%) patients in the CA+AMI group would qualify for ICD implantation.

At the time of admission to the hospital, there was evidence on the surface electrocardiogram of ST segment elevation AMI in 16 of 48 (33.3%) patients in the concurrent CA+AMI group compared to 52 of 96 (54.2%) in the AMI group (p=0.014). Of the 144 patients in the CA+AMI and AMI groups, 33 (16%) underwent revascularization (12 by percutaneous coronary intervention and 11 by coronary artery bypass grafting) at the time of the index hospitalization or during follow-up.

### Time to Death or First Ventricular Arrhythmia

**Figure 1:**
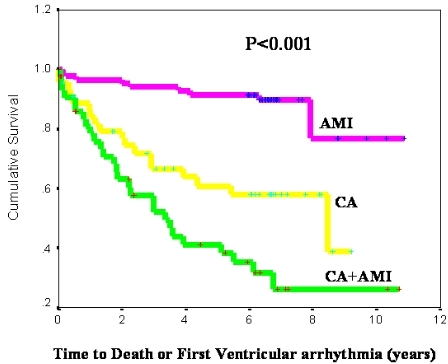
Time to death or first ventricular arrhythmia by study group

We compared the time to death or first VA in the 3 patient study groups. Patients in the CA+AMI group had lower VA-free survival compared to patients in the control groups (5-year VA-free survival of 67%, 92%, and 80% for patients in the CA+AMI, AMI, and CA groups respectively, p<0.001). Kaplan Meier curves were constructed to reflect the VA-free survival over the mean follow-up period of 3.9±3.2 years and are shown in Figure 1.

The VA-free survival was analyzed in a multivariate Cox Regression model adjusting for such covariates as age, LVEF, QRS width, cardiac rhythm documented at the time of hospital admission, and implementation of therapies such as coronary revascularization, use of β-blockers, use of anti-arrhythmic drugs, and defibrillator implantation. After adjusting for all these covariates, patients with concurrent CA and AMI remained at a higher risk compared to the 2 control groups for death or recurrent VA (Hazard Ratio, HR=3.3, 95% confidence interval =1.4-8.1, adjusted p=0.008).

### Incidence of Ventricular Arrhythmia

**Figure 2:**
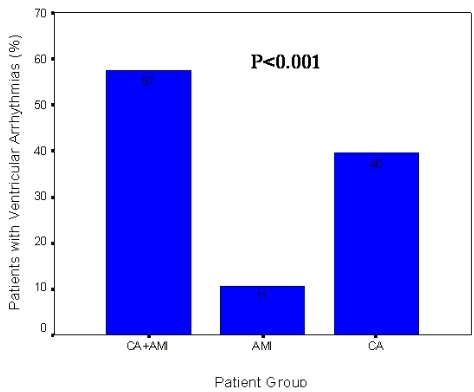
Incidence of death or ventricular arrhythmia by study group

Patients with concurrent CA and AMI also had a higher incidence of VA when compared to patients with CA without AMI and patients with AMI without CA. The incidence of VA recurrence over the follow-up period was 57%, 40% and 11% in the CA+AMI, CA, and AMI groups, respectively (p<0.001). The incidence of VA among the 3 study groups is shown in Figure 2.

The risk of VA was also analyzed using a multivariate binary logistic regression model. After correcting for age, LVEF, QRS width, cardiac rhythm documented at hospital admission, coronary revascularization, utilization of β-blockers, anti-arrhythmic drugs, and ICD implantation, patients with concurrent CA and AMI remained at a higher risk for VA recurrence (HR=5.1, 95% confidence interval =1.4-19.1, adjusted p=0.015).

### Time to Death Among Study Groups and Effect of Appropriate ICD Therapy

We analyzed the time to death in the 3 study groups. In the overall cohort, there was no difference in survival (p=0.75, Figure 3a). Because a large proportion of patients in our study had defibrillators and given the established mortality benefits conferred by the ICD, we conducted analysis on the cumulative incidence of appropriate ICD therapies, defined as ICD shocks or anti-tachycardia pacing events for treatment of VA, in the subgroup of patients who had an ICD (73%, 7%, and 98% of patients in the CA+AMI, AMI, and CA groups, respectively). The total number of patients implanted with an ICD was 89 (46% of the overall population). As shown in Figure 3b, the time to first appropriate ICD therapy was significantly shorter in the group of patients with concurrent CA and AMI or CA alone compared to the AMI group (2-year cumulative incidence of appropriate ICD therapy of 36%, 34%, and 0% for the CA+AMI, CA, and AMI groups respectively, p<0.001).

## Discussion

Our data demonstrate that patients with concurrent CA and AMI are at significantly increased risk of death or recurrent VA compared to patients with AMI and no associated CA. Also, their risk exceeds that of patients with CA outside the immediate context of an AMI, a population that has an established indication for ICD implantation according to current published guidelines4 which are based on a number of large, randomized, prospective trials [1-3]. This finding is consistent with prior published data [5,11] where patients with reversible causes of CA or VA were found to have a high mortality rate comparable to that of patients randomized to the control (no ICD) arm in the AVID trial [1]. Although there was no difference in total mortality between the 3 groups in our study, this is probably accounted for by the difference in ICD implantation and appropriate ICD therapy delivered amongst the 3 groups. Although not every shock translates into a life saved [12], almost every life saved by a defibrillator is from an appropriate ICD therapy. This is a plausible statement, given the documented mode of death in survivors of CA who do not receive an ICD1 and given the intrinsic mode of function of the ICD.

While current guidelines support the implantation of an ICD for patients with cardiomyopathy on the basis of persistently depressed left ventricular function and even in the absence of previous arrhythmic events [8,9], survivors of concurrent CA and AMI do not qualify for ICD therapy [4]. Our study suggests that patients with concurrent CA and AMI are at a similar mortality risk and at a higher risk for recurrent VA compared to CA survivors. Hence, ICD implantation among this patient population should be considered. Also, with the expanding indications for ICD implantation for primary prevention [6,9], many survivors of CA in the context of an AMI would have an indication for ICD implantation anyway, albeit few months after the event. This fact may need to be factored into the decision making about early ICD implantation, prior to hospital discharge.

Some studies [13] have questioned the value of the ICD implantation early after AMI. The DINAMIT study [13] demonstrated that early ICD therapy is associated with a reduction in the rate of arrhythmic deaths but that this benefit was offset by an increase in the rate of death from non-arrhythmic causes.  It is important however to keep in mind the fact that DINAMIT enrolled patients whose ICDs were implanted for primary prevention purposes as opposed to our current study which deals with survivors of a CA. Even in the context of primary prevention, the risks of waiting before implanting an ICD is challenged by the high risk of CA early after AMI that has been demonstrated in the VALIANT study [14].

Mechanistically, denying a life-saving therapy such as the ICD to patients with reversible causes of VA or CA is flawed for 2 reasons. First, patients who suffer a CA secondary to presumed reversible causes such as ischemia, myocardial injury, or electrolyte imbalances are at continued risk of recurring episodes of such reversible, albeit not avoidable, events. Second, The CA that happens secondary to a reversible cause may just reflect an underlying predisposition, genetic or other, in the survivor of such event i.e. a different trigger in the same subject may lead to a future CA because of a lower fibrillation threshold that the patient may have. Although speculative, these concerns are very plausible and have to be considered before denying a patient an ICD implantation because their CA was concurrent with an AMI.

Our study has some limitations. First, the data was collected retrospectively and therefore its accuracy depends on the accuracy of the electronic medical records and clinical charts. Some patients who are not currently followed at our institution might have had VA after they were lost to follow-up, which could not be accounted for. In addition, patient compliance with standard medical therapy after hospital discharge could not be ascertained. Second, our study was a single center study with a moderate number of patients who were mainly male Caucasians. Extrapolating our results to other patient populations from different ethnic or geographic backgrounds may not be attainable.

In summary, our data suggests that survivors of concurrent CA and AMI are at high risk for death and recurrent VA. While in the current guidelines these patients are denied ICD implantation, our study suggests that they may benefit from this life-saving therapy. Further prospective, randomized studies are however needed to establish the potential effect of the ICD on the overall mortality of survivors of concurrent CA and AMI before the current guidelines may be revised.

## Figures and Tables

**Figure 3a:**
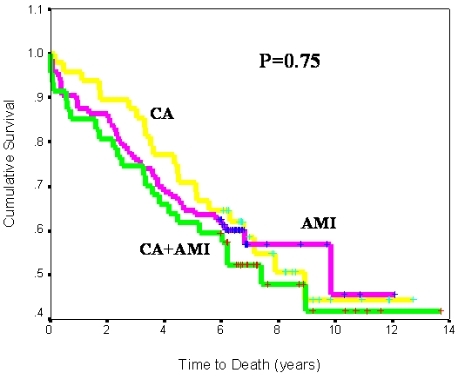
Time to death by study group

**Figure 3b:**
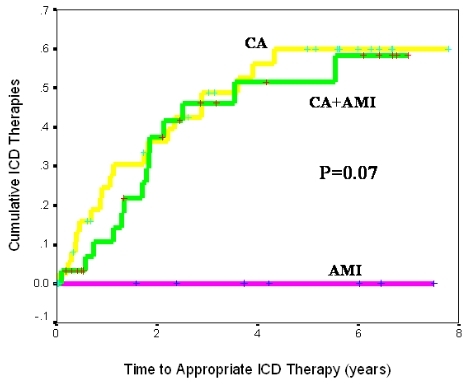
Cumulative incidence of appropriate ICD therapy by study group

**Table 1:**
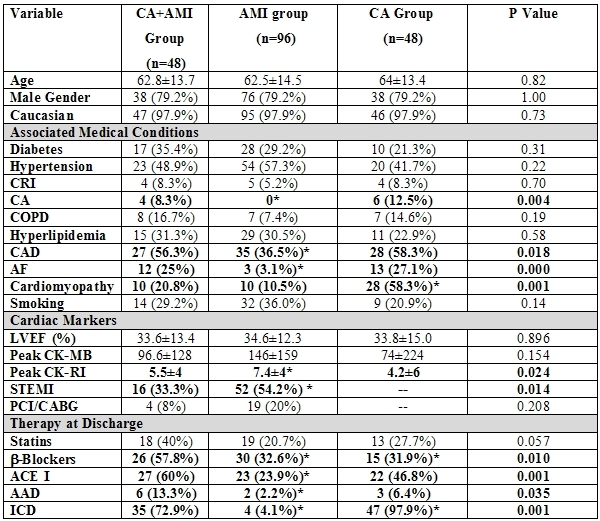
General demographic and clinical characteristics of the study population.

CA=cardiac arrest; AMI=acute myocardial infarction; CRI=chronic renal insufficiency; COPD=chronic obstructive pulmonary disease; CAD=coronary artery disease; AF=atrial fibrillation; LVEF=left ventricular ejection fraction; CK-MB=creatinine kinase-MB fraction; CK-RI= creatinine kinase-risk index; STEMI=ST segment elevation myocardial infarction; PCI/CABG=percutaneous coronary intervention/coronary artery bypass grafting; ACE I=angiotensin converting enzyme inhibitor; AAD=anti-arrhythmic drug; ICD=Implantable Cardioverter-defibrillator. (*) p≤ 0.05 for comparisons with the CA+AMI group of patients.

## Abbreviations

AMI: acute myocardial infarction
CA: cardiac arrest
CAD: coronary artery disease
HR: hazard ratio
ICD: implantable cardioverter-defibrillator
LVEF: left ventricular ejection fraction
VA: ventricular arrhythmia
